# Trends in Mechanical Intestinal Obstruction: A 30‑Year Comparative Analysis Between Developing and Developed Nations

**DOI:** 10.5334/aogh.4782

**Published:** 2025-12-16

**Authors:** Abdel Rahman Al Manasra, Alyaman Mohammad, Hamzeh Alsamarah, Leen Alshobaki, Shefaa Alenezi, Ula Altorman, Tameem Shotar, Salma Alrousan, Anas Aljaiuossi

**Affiliations:** 1 King Abdullah university Hospital, Jordan university of science and technology, Irbid, Jordan; 2 Jordan university of science and technology, Irbid, Jordan; 3 Yarmouk university, Irbid, Jordan

**Keywords:** mechanical intestinal obstruction, adhesions, global surgery, developing countries, surgical outcomes, Jordan

## Abstract

*Background:* Mechanical intestinal obstruction (MIO) remains a global surgical emergency with significant morbidity and mortality. While prior studies suggest divergent etiological patterns between developed and developing nations, recent trends and underlying drivers remain understudied.

*Objectives:* This study evaluates 30‑year trends in MIO etiology and outcomes in Jordan and compares these with global data to assess convergence of patterns.

*Methods:* A retrospective cohort study (2020–2023) of MIO patients at a tertiary Jordanian center was combined with a systematic literature review of MIO etiologies in developed and developing nations pre‑ and post‑2000. Data included demographics, management, outcomes, and mortality. Statistical analysis employed chi‑square and t‑tests.

*Findings:* Postoperative adhesions were the leading cause of MIO (64% in 2023, rising from 25% in 1993), followed by malignancy (20%) and hernias (5%). Mortality was 10%, with sepsis as the primary cause. Global comparisons revealed adhesions as the predominant etiology in both developed (7/9 studies, 77.7%) and developing nations (8/14 studies, 57.1%) post‑2000, contrasting with historical hernia predominance in developing regions.

*Conclusion:* Adhesions have become the leading cause of MIO globally, reflecting increased surgical access and aging populations. Socioeconomic advancements in developing nations may explain converging trends with developed countries. Standardized global reporting and adhesion prevention strategies are urgently needed.

## 1. Introduction

Mechanical intestinal obstruction (MIO) is a life‑threatening surgical condition accounting for 20% of acute abdominal emergencies worldwide [1]. Traditional narratives attribute MIO etiology to divergent patterns: postoperative adhesions dominate in developed nations, while obstructed hernias prevail in developing regions due to limited surgical access [2, 3]. However, recent evidence suggests these distinctions may be fading, driven by improved healthcare infrastructure and rising surgical volumes in developing economies [4, 5].

Jordan’s healthcare system, a model for rapid development in the Middle East, provides an ideal setting to investigate these shifts. Over the past three decades, Jordan has achieved > 90% hospital delivery rates and expanded laparoscopic surgery access [6], yet no studies have examined how these changes impact MIO patterns.

We aimed to analyze 30‑year trends in MIO etiology and outcomes in Jordan and to compare these trends with global data to assess convergence between developed and developing nations. We also intend to identify modifiable drivers of disparities, including socioeconomic and cultural factors.

By addressing these aims, we contribute to the World Health Organization’s Global Surgery 2030 agenda [7] and inform targeted interventions for MIO prevention.

## 2. Methodology

### 2.1 Study design

This study employed a dual approach combining retrospective cohort analysis with a systematic literature review. Data were collected for patients aged ≥ 13 years diagnosed with MIO at King Abdullah University Hospital (KAUH), Jordan, between 2020 and 2023. The hospital is the largest medical structure in the north of the country, serving nearly two million citizens from four governorates and being a good source for a representative sample.

### 2.2 Inclusion and exclusion criteria

Inclusion criteria required confirmation of MIO through imaging or surgical findings in patients aged 13 or older who were admitted and managed at KAUH between 2020 and 2023. Patients were excluded if they had nonmechanical causes of intestinal blockage, such as electrolyte imbalance, paralytic ileus, or pseudo‑obstruction, or if they were 12 years old or younger.

### 2.3 Data preparation

The datasets used in this research were gathered by reviewing electronic as well as paper‑based medical records. The following clinical and demographic data were extracted after reviewing each patient’s chart: gender, age, date of admission, main presenting symptom, associated symptoms, duration of symptoms, duration of hospitalization, intensive care unit (ICU) admission, comorbidities, past surgical history, the etiology of MIO, management, and outcomes.

**Figure 1:**
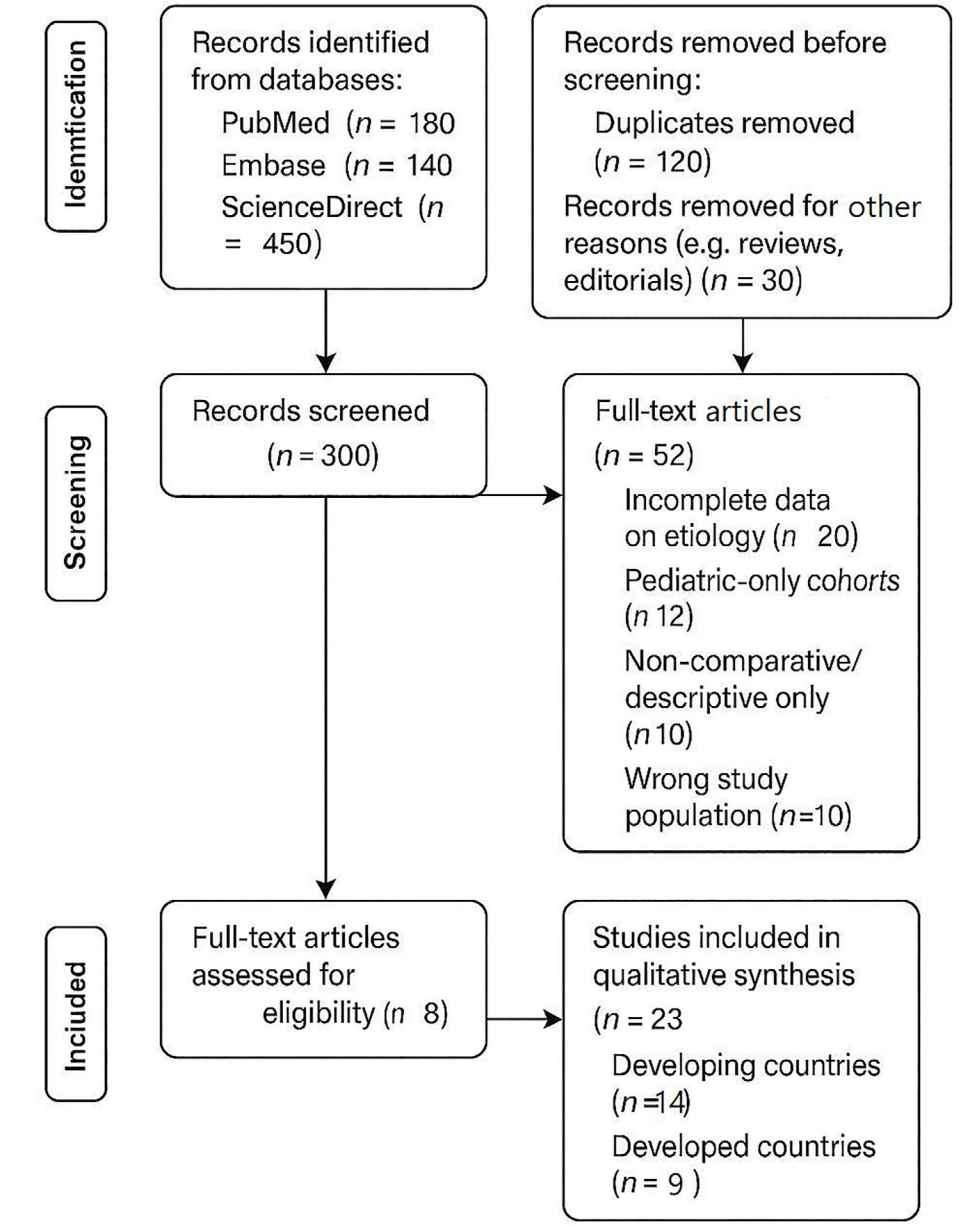
PRISMA 2020 flow diagram for the systematic review of MIO etiology (pre‑and post‑2000).

For the global comparison, we conducted a systematic search of PubMed, Embase, and ScienceDirect databases for studies published before and after 2000, focusing on reports detailing MIO etiology and outcomes from both developed and developing nations. The literature search followed Preferred Reporting Items for Systematic Reviews and Meta‑Analyses (PRISMA) guidelines, with particular attention to studies providing comparative data across different economic settings; Figure 1 shows the PRISMA 2020 flow diagram for the systematic review of MIO etiology (pre‑and post‑2000). The year 2000 was used as a cutoff point since it has been considered a transition for advancement in medical technologies, including imaging modalities, and the beginning of the widespread use of the internet in healthcare for research and communication. Moreover, in the late 1990s and early 2000s, clinical practice shifted to utilize minimally invasive techniques in surgery and relied more on the rising evidence‑based medicine and data from systematic research. Statistical analysis was performed using SPSS version 26, with categorical variables expressed as percentages and continuous variables as means ± standard deviation.

### 2.4 Definitions

For study purposes, MIO was defined as a blockage in the intestine that is caused by an intrinsic or extrinsic physical barrier [1].

The Simplified Acute Physiology Score (SAPS) III score was utilized in this study as a validated tool that can accurately predict mortality risk in patients admitted to critical care units [8].

We used the statistical annex from the 2024 World Economic Situation and Prospects (WESP) report to classify countries. The statistical annex includes data used by the WESP to identify trends in many aspects of the global economy. It was created by the Economic Analysis and Policy Division (EAPD) of the Department of Economic and Social Affairs at the United Nations Secretariat. The WESP report classifies countries into one of three broad categories: developed, economies in transition, and developing economies [9].

### 2.5 Ethical considerations

Patients’ confidentiality was protected in accordance with the Declaration of Helsinki provisions. This study was approved by the ethics committee at our institution (reference number: 20230279).

## 3. Results

The study cohort comprised 177 patients with a mean age of 54.4 ± 19 years, of whom 55% were male. Table 1 demonstrates the baseline characteristics of patients with MIO.

**Table 1:** Baseline characteristics and clinical outcomes of patients with mechanical intestinal obstruction (n = 177).

VARIABLE	VALUE
Mean age (years)	54.4 ± 19 (Range: 13–81)
Gender	Male: 97 (55%) Female: 80 (45%)
Presenting symptoms	Vomiting: 139 (79%) Abdominal pain: 136 (77%) Constipation: 98 (56%) Nausea: 46 (26%)
Comorbidities	Hypertension: 67 (39%) Diabetes: 45 (26%) Malignancy: 43 (25%) Heart failure: 13 (7%) IBD: 9 (5%)
Previous abdominal/pelvic surgery	142 (81%)
Mean duration of symptoms (days)	6.5 ± 4.7 (Range: 1–60)
Mean hospital stay (days)	5.6 ± 3.5 (Range: 1–30)
ICU admissions	15 (8%)
SAPS III score^a^ (ICU patients)	Mean: 62 (Range: 42–76)
ICU mortality	6/15 (40%)
Overall mortality (all patients)	18 (10%)
Causes of death	Sepsis: 17 (94%) Aspiration pneumonia: 1 (6%)
Outcomes (within 3 months)	Full recovery: 119 (68%) Recurrence: 30 (17%) Death: 18 (10%) Complications (incl. re‑op): 7 (4%)

a: SAPS: Simplified acute physiologic score, for patients admitted to ICU.

Postoperative adhesions were identified as the leading cause (64%), followed by gastrointestinal malignancies (20%) and obstructed hernias (5%). Less common causes included inflammatory bowel disease (3%), sigmoid volvulus (1.5%), and diverticular disease (1%). Conservative therapy was effective in 64% of cases, while 36% warranted surgical intervention, primarily adhesiolysis (37% of procedures). Tables 2a and 2b summarize etiology and management modalities of MIO.

**Table 2a:** Etiology of mechanical intestinal obstruction (n = 177).

ETIOLOGY	NO. OF PATIENTS (%)
Adhesions	112 (64%)
Malignancy	36 (20%)
Hernia	9 (5%)
Inflammatory bowel disease (IBD)	7 (3%)
Sigmoid volvulus	3 (1.5%)
Diverticular disease	2 (1%)
Intussusception	2 (1%)
Malrotation	2 (1%)

**Table 2b:** Management modalities and surgical procedures.

MANAGEMENT APPROACH/SURGICAL PROCEDURE	NO. OF PATIENTS/CASES
Conservative treatment	113 (64%)
Surgical intervention	62 (36%)
Adhesiolysis	23 (37%)
Colectomy	16 (26%)
Small bowel resection	4 (6%)
Colostomy	4 (6%)
Sigmoidectomy (Hartmann’s)	3 (5%)
Ileostomy	1 (1.6%)
Ladd’s procedure	2 (3%)
Hernia repair	2 (3%)
Meckel diverticulectomy	1 (1.6%)
Gastrojejunostomy	1 (1.6%)

The overall mortality rate was 10%, but increased to 40% among ICU‑admitted patients (n = 15), with sepsis accounting for 94% of deaths. SAPS III scores for ICU patients averaged 62 (range 42–76), with a predicted mortality rate of 41%. The main clinical outcomes are illustrated in Table 1, as well.

**Figure 2:**
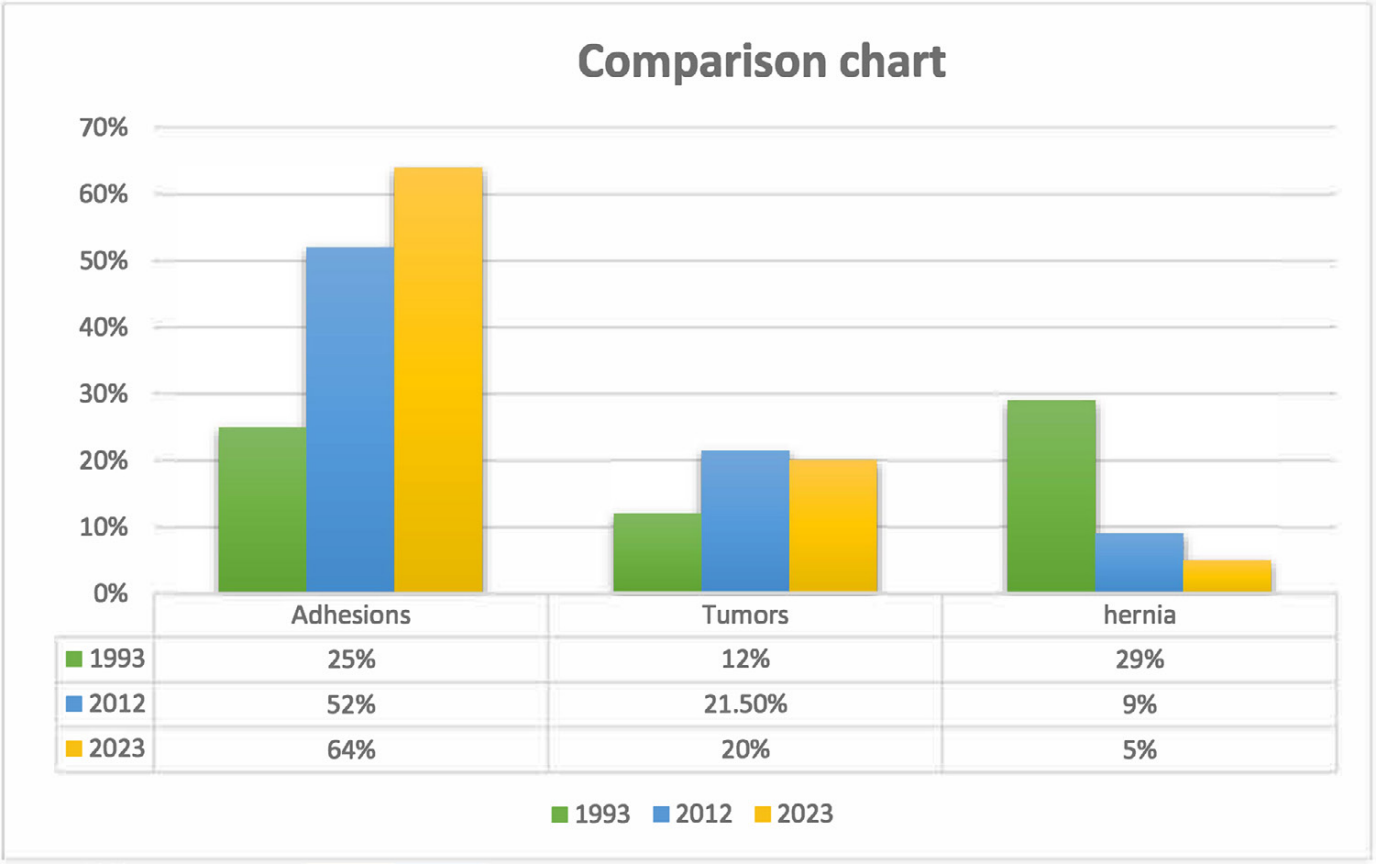
Percentage of the main causes of MIO in North Jordan (1993, 2012, and 2023).

Comparative analysis of global data revealed adhesions as the leading cause of MIO in both developed (85.7% of post‑2000 studies) and developing nations (57.1%), though hernia‑related obstructions remained more prevalent in low‑resource settings (28.6% in post‑2000 studies versus 42.9% pre‑2000). Longitudinal data from Jordan demonstrated a striking increase in adhesion‑related MIO, from 25% in 1993 to 64% in 2023, paralleling increased access to abdominal surgeries and laparoscopic procedures. Figure 2 demonstrates the main causes of mechanical bowel obstruction in North Jordan between 1993 and 2023. Notably, recurrence rates were higher in surgically managed patients (17%) compared to those treated conservatively (12%). Table 3 demonstrates a global comparison of MIO etiology and mortality by region and time period.

**Table 3:** Global comparison of MIO etiology and mortality by region and time period.

STUDY (YEAR)	COUNTRY	PERIOD	ECONOMIC STATUS	MOST COMMON CAUSE	MORTALITY RATE
Ti et al. (1976) [11]	Malaysia	5 yrs pre‑2000	Developing	Adhesions	9–13%
Pal et al. (1982) [12]	India	5 yrs pre‑2000	Developing	Ventral hernias	28%
Steityeh et al. (1993) [4]	Jordan	pre‑2000	Developing	Ventral hernias	NA
Hasnain et al. (1994) [2]	Pakistan	1987–1991	Developing	Adhesions 43%	NA
Mohamed et al. (1997) [13]	Saudi Arabia	10 yrs pre‑2000	Developing	Adhesions 45%, then hernia	3.5%
Ntakiyiruta & Mukarugwiro (2009) [14]	Rwanda	2003–2007	Developing	Ventral hernias	NA
Obaid (2011) [15]	Malaysia	2003–2007	Developing	Ventral hernias	4.3%
Kapan et al. (2012) [16]	Turkey	2005–2010	Developing	Adhesions	NA
Jiang et al. (2019) [17]	China	2004–2013	Developing	Adhesions	NA
Yusuf et al. (2014) [18]	Pakistan	2012–2013	Developing	Strictures	NA
Jena et al. (2021) [19]	India	1996–2019	Developing	Adhesions ↑ from 23% to 51.6%	NA
Fekadu et al. (2022) [20]	Ethiopia	2022	Developing	Small bowel volvulus	NA
Idrobo et al. (2020) [21]	Colombia	2012–2013	Developing	Adhesions	1%
Wasim Yusuf et al. (2014) [18]	Pakistan	2012	Developing	Obstruction/perforation (histopathology)	NA
Tondelli et al. (1983) [22]	Switzerland	5 yrs pre‑2000	Developed	Adhesions	14%
Cross & Johnston (1987) [23]	Ireland	1947–1982	Developed	Ventral hernia	NA
da Silva et al. (1994) [24]	Portugal	1981–1991	Developed	Hernia 44%, then adhesions 15%	10.8%
Miller et al. (2000) [25]	Canada	1986–1996	Developed	Adhesions (then Crohn’s)	NA
Markogiannakis et al. (2007) [26]	Greece	2001–2002	Developed	Adhesions	0.8%
Stephenson & Singh (2011) [27]	UK	2011	Developed	Adhesions	10–25%
Trivedi et al. (2012) [28]	USA	2005–2011	Developed	Adhesions	NA
Beardsley et al. (2014) [29]	Australia	2009–2013	Developed	Adhesions	NA
Paul et al. (2022) [30]	Germany	2009–2019	Developed	Adhesions	4.9–15.9%

## 4. Discussion

Our center’s data have shown that over the past 30 years, adhesions have become more prevalent as a cause of intestinal obstruction, rising from 25% in 1993 to 52% in 2012 up to 64% in 2023, as shown in Figure 2. Data from developed and developing countries revealed similar predominance of adhesions as a leading cause of MIO prior to and after 2000, as also shown in Table 3 [3–5].

Many authors argued that hernias are more predominant as a cause of MIO in developing countries, accounting for socioeconomic status and hence the shortage in medical resources. In populations with poor economic status, lack of access to healthcare services and thereby delayed presentation, as well as the inability to quantify the danger of delayed hernia repair, were blamed for hernia being a leading cause of MIO in these communities [3, 10, 31, 32].

In fact, several factors may explain why adhesions have become a more predominant cause of MIO; these include the considerable rise in the number of abdominal surgeries performed—including transabdominal hernia repairs—which can all lead to the formation of adhesions. In other words, it is our surgical interventions for abdominal diseases that resulted in a large increase in the number of MIOs [10, 11, 33, 34].

Furthermore, in many countries, individual life expectancy has grown, and elderly adults are more likely to have had many abdominal procedures, which increases the cumulative risk of developing adhesions over time [10].

It is worth noting that many hernias (mainly inguinal) complicated by MIO are classified upon admission as complicated hernias rather than MIO—particularly where Current Procedural Terminology (CPT) coding is not used—which results in fewer hernias being held accountable for intestinal blockage. Moreover, the success in managing hernias shifts the statistical balance, making adhesions appear more significant as a cause of obstruction [34].

Other factors that may also contribute to the variation in causes of MIO between nations include ethnic backgrounds. However, data regarding racial disparities are scarce. In a study from Malaysia, 261 patients from different ethnic groups were operated on between 1968 and 1972 for MIO. The pattern of intestinal obstruction in Chinese was similar to that in Caucasians, where adhesions account for the largest number of cases. This pattern was ascribed to the high rate of abdominal operations performed on Chinese who commonly suffer from peptic ulcers, gastric cancers, colon cancer, and other pathologies that need surgeries. The occurrence in Malays, Indians, Pakistanis, and Ceylonese was comparable to that in other developing countries where external hernia is commonest; this was explained by two reasons: cultural, which makes surgery not readily acceptable, and economic, which makes surgery unavailable as a medical service [34].

Before 2000, patients in Jordan typically did not seek surgical intervention as their first option for treating surgical conditions. A valid cultural belief that “the last remedy is cauterization” emphasized the preference for exhausting all conservative treatments before considering surgery. However, misuse of this approach was associated with a higher incidence of complicated hernias presenting with MIO. Over recent decades, local and global advancements in healthcare services and education, coupled with heightened public awareness about the significance of promptly seeking medical attention, have influenced the management and outcomes of health issues. There seems to be an increased awareness toward elective hernia repair, which may have led to a decline in the incidence of hernia‑related bowel obstructions yet an increase in the proportion of postoperative adhesions [4, 5].

Minimally invasive abdominal surgeries have become increasingly widespread over the past 20–30 years. Although studies have consistently shown that minimally invasive abdominal procedures are associated with a lower incidence of adhesion formation compared to conventional open techniques, adhesions have remained on top of the list as a leading cause for mechanical bowel obstruction. This is likely explained by the complex process of adhesion formation, which is affected by multiple other factors, similar to genetic predisposition, the extent of manipulation and handling of the intestine, and the individual healing characteristics [34, 35].

This study had some limitations. First, it was a retrospective design and involved data from a single center. Second, the sample size of patients with MIO was relatively small to evaluate risk factors with statistical significance. Further multi‑center prospective studies and meta‑analyses are required to better investigate variations in patterns between different countries and validate trends.

## 5. Conclusion

Adhesions have become the leading cause of MIO globally, reflecting increased surgical access and aging populations. Socioeconomic advancements in developing nations may explain the converging trends with developed countries. Standardized global reporting and adhesion prevention strategies are urgently needed. Despite the improvement in diagnosis and management of MIO, the mortality rate associated with MIO remains relatively high. Some MIO cases are inherently severe, and even with awareness, timely management may not always prevent adverse outcomes. Older patients with comorbidities face higher risks, contributing to mortality rates.

## Abbreviations

MIO = mechanical intestinal obstruction, MR = mortality rate, KAUH = King Abdullah University Hospital, ICU = intensive care unit, SAPS = Simplified Acute Physiology Score, EAPD = Economic Analysis and Policy Division, IBD = inflammatory bowel disease, WESP = World Economic Situation and Prospects, CPT = Current Procedural Terminology.
